# Phosphatidylinositol-Glycan-Phospholipase D Is Involved in Neurodegeneration in Prion Disease

**DOI:** 10.1371/journal.pone.0122120

**Published:** 2015-04-13

**Authors:** Jae-Kwang Jin, Byungki Jang, Hyoung Tae Jin, Eun-Kyoung Choi, Cha-Gyun Jung, Hiroyasu Akatsu, Jae-Il Kim, Richard I. Carp, Yong-Sun Kim

**Affiliations:** 1 Ilsong Institute of Life Science, Hallym University, Anyang, Gyeonggi-do 431–060, Korea; 2 Department of Neurophysiology and Brain Science, Nagoya City University Graduate, School of Medical Sciences, Nagoya, Aichi 467–8601, Japan; 3 Choju Medical Institute, Fukushimura Hospital, Toyohashi 441-8124, Japan; 4 Department of Food Science and Nutrition, Pukyong National University, Busan 608–737, Korea; 5 Department of Virology, New York State Institute for Basic Research in Developmental Disabilities, Staten Island, NY 10314, United States of America; Van Andel Institute, UNITED STATES

## Abstract

PrP^Sc^ is formed from a normal glycosylphosphatidylinositol (GPI)-anchored prion protein (PrP^C^) by a posttranslational modification. Most GPI-anchored proteins have been shown to be cleaved by GPI phospholipases. Recently, GPI-phospholipase D (GPI-PLD) was shown to be a strictly specific enzyme for GPI anchors. To investigate the involvement of GPI-PLD in the processes of neurodegeneration in prion diseases, we examined the mRNA and protein expression levels of GPI-PLD in the brains of a prion animal model (scrapie), and in both the brains and cerebrospinal fluids (CSF) of sporadic and familial Creutzfeldt-Jakob disease (CJD) patients. We found that compared with controls, the expression of GPI-PLD was dramatically down-regulated in the brains of scrapie-infected mice, especially in the caveolin-enriched membrane fractions. Interestingly, the observed decrease in GPI-PLD expression levels began at the same time that PrP^Sc^ began to accumulate in the infected brains and this decrease was also observed in both the brain and CSF of CJD patients; however, no differences in expression were observed in either the brains or CSF specimens from Alzheimer’s disease patients. Taken together, these results suggest that the down-regulation of GPI-PLD protein may be involved in prion propagation in the brains of prion diseases.

## Introduction

Prion diseases are a group of neurodegenerative disorders that affect the central nervous system in humans and animals. Creutzfeldt-Jakob disease (CJD) and scrapie are an archetype of the group of neurological diseases referred to as prion disease or spongiform encephalopathy, and is characterized histologically by vacuolation and astrocytosis in the brains of humans, sheep and goats [1, 2]. The etiological agent of prion disease is PrP^Sc^, which is an abnormal isoform that is converted from the normal cellular protein PrP^C^ by unknown posttranslational modification processes [3]. PrP^C^ is synthesized in the endoplasmic reticulum, enters into caveolae-like domains (CLDs) and is bound to the plasma membrane by a glycophosphatidylinositol (GPI) anchor [4]. The CLDs are rich in cholesterol and glycosphingolipids and, contain many GPI-anchored proteins including PrP^C^ [5–7]. In a previous study, cholesterol depletion was reported to inhibit PrP^Sc^ formation in the sphingolipid-cholesterol-rich compartment [5]. In contrast, sphingolipid depletion increased PrP^Sc^ formation in neuroblastoma cells infected with a prion agent [8]. Furthermore, several lines of evidence have suggested that CLDs are sites for the generation of PrP^Sc^ [9]. The posttranslational conversion of PrP^C^ into the scrapie isoform of PrP^C^ (PrP^Sc^) is a peculiar feature among the pathophysiological observations in prion diseases [10]. Despite an increased knowledge of the posttranslational prion conversion, the exact mechanisms of the conversion have not been fully elucidated.

The GPI-anchored proteins can be removed from the cell surface by phosphatidylinositol-glycan-specific phospholipase. Thus, we speculated that, in prion disease, a conformational change in the anchored normal prion isoform, PrP^C^, may be directly or indirectly induced by the suppression of phosphatidylinositol-glycan-specific phospholipase activity. Most previous studies on the GPI metabolism of GPI-anchored proteins have focused on the phosphatidylinositol-specific phospholipase C (PI-PLC). However, very little is known regarding the role of phosphatidylinositol-glycan-specific phospholipase D (GPI-PLD), which is a specific enzyme for GPI anchors. There are low-reactivity antibodies to rodent GPI-PLD, and progress in this area is inhibited by the lack of a high titer-specific antibody.

GPI-PLD, which is abundant in mammalian serum, is a 110- to 120-kDa N-glycosylated protein and is a high-density lipoprotein-associated protein [11, 12]. This enzyme has been extensively studied in many cells including hepatocytes, pancreatic islets [13] and macrophages [14]. The liver appears to be the primary source of circulating GPI-PLD [15]. However, no studies on the physiological and pathological function have been reported in the brain. Because a GPI-anchored protein, PrP^C^, is known to be a major factor in scrapie pathology, the exact role of GPI-PLD must be clarified in the brains of CJD patients including a prion animal model.

In the present study we investigated whether GPI-PLD expression is changed during the process of neurodegeneration in prion diseases, and we speculated on the possible involvement of GPI-PLD in the conversion of PrP^C^ during the neurodegenerative process of prion disease.

## Materials and Methods

### Antisera

The following monoclonal antibodies and polyclonal antisera were used; mouse anti-PrP 10E4 (kindly provided by Dr. Richard Rubenstein in New York State Institute for Basic Research, Staten Island, NY, USA), mouse anti-caveolin1, mouse monoclonal anti-β-actin (Transduction Laboratories, Lexington, KY), rabbit anti-14-3-3, goat anti-GPI-PLD (Santa Cruz Biotechnology, Santa Cruz, CA), and rabbit anti-GFAP (Dako, Copenhagen, Denmark). Non-immune serum for immunohistochemical analyses was obtained from Jackson ImmunoResearch Laboratories (West Grove, PA).

### Animals and the scrapie strain

C57BL mice were purchased from the Jackson Laboratory (Bar Harbor, ME) and knockout mice in which the Prnp gene was deleted (PrP knockout, Zurich) were obtained from Dr. Adriano Aguzzi, University of Zurich. The mice were bred and maintained in an animal facility in Hallym University. The ME7 scrapie strain was kindly provided by Dr. Alan Dickinson of the Neuropathogenesis Unit (Edinburgh, Scotland, UK). The animals were inoculated intracerebrally with 30 μl of 1% (w/v) brain homogenates in 0.01 M phosphate-buffered saline (PBS) prepared from an ME7-infected C57BL mouse. Control mice were injected with 30 μl of 1% (w/v) homogenates of a normal mouse brain and harvested at the same age as the scrapie-positive mice. Laboratory animal experiments were approved by the Hallym Medical Center Intuition Animal Care and Use Committee. The protocol animal handling was accordance with institutional and international guidelines.

### Creutzfeldt-Jakob disease (CJD) and Alzheimer’s disease (AD) samples

The brain and cerebral spinal fluid (CSF) specimens from CJD patients and age-matched controls were obtained from the CJD Autopsy Center (Hallym University Sacred Heart Hospital, Korea). A total of four post-mortem brain specimens from AD patients and age-matched controls were provided by Dr. Piotr Kozlowski (New York State Institute for Basic Research in Developmental Disabilities, Staten Island, NY 10314, USA), and five CSF samples were obtained from AD patients at Fukushimura Hospital (Toyohashi, Japan). The CSF was frozen immediately in liquid nitrogen at the site of the lumbar tap and stored at -70°C until use. Experiments using AD CSF were performed after obtaining informed consent from the patients’ guardians to use the CSF samples for diagnosis and research, including biochemical, molecular biochemical, and genomic analyses. The characteristics of the brain specimens with post-mortem intervals ranging from 0.5 to 120 h are shown in Tables 1 and 2. The cases of CJD and AD were classified based on the quantitative pathological and physiological features including PrP^Sc^, 14-3-3 protein, senile plaques, and neurofibrillary tangles (NFT), according to the criteria suggested by Braak and Braak [16]. The study was approved by the Institutional Review Board at Hallym University.

**Table 1:** Demographic details of post-mortem brain specimens from controls and Creutzfeldt-Jakob disease patients.

No.	Case type	Age/sex	Post-mortem interval (h)	Brain weight (g)
1	Control	83/M	12.0	1,220
2	Control	67/F	4.0	1,400
3	Control	71/M	7.0	1,225
4	Sporadic CJD	77/M	2.5	1,600
5	Sporadic CJD	49/M	120.0	1,150
6	Sporadic CJD	66/F	13.0	1,380
7	Familial CJD	66/F	13.5	1,450

**Table 2:** Demographic details of post-mortem brain specimens from controls and Alzheimer’s disease patients.

No.	Case type	Age/sex	Post-mortem interval (h)	Brain weight (g)	Braak stage of AD brains [15]
1	Control	68/F	2.5	1,240	None
2	Control	68/F	5.0	1,200	None
3	Control	67/F	4.0	1,400	None
4	AD	74/F	14.5	1,150	III/IV
5	AD	79/F	5.0	1,090	III/IV
6	AD	73/F	0.5	1,210	III/IV
7	AD	76/M	7.5	1,125	III/IV

### Preparation of brain tissues

Brains from both control and scrapie-injected mice were harvested at 60 to 160 days post-infection (dpi). The mice were perfused transcardially with PBS for Western blot analysis. For the reverse transcription–polymerase chain reaction (RT–PCR), the brains were removed immediately from anesthetized mice and stored at -70°C until analysis.

### Reverse transcription (RT)-polymerase chain reaction (PCR)

Total RNA samples from whole mouse brains (sampled at 160 dpi) were extracted with TRIzol reagent (Gibco-BRL, Rockville, MD), according to the manufacturer’s instructions. RT–PCR was performed as follows. The cDNA was synthesized from 2 μg of total RNA by RT using AMV reverse transcriptase (Promega, Madison, WI) and oligo (dT) primer. After incubation for 1 h at 42°C, the samples were heat-inactivated and kept at 4°C. A 5 μl aliquot of the cDNA of each sample was used for PCR with the GPI-PLD specific primers. The PCR conditions consisted of an initial denaturation step at 94°C for 2 min, then 30 cycles at 94°C for 1 min, 59°C for 1 min, and 72°C for 1.5 min, with a final extension at 72°C for 6 min. The PCR products were separated on a 1.5% agarose gel and visualized with ethidium bromide staining under UV light. The cDNA was also amplified with a primer pair for the housekeeping gene actin. The PCR primers used were 5'-ATTTTGGAGGAGATGTGTTG-3' (GPI-PLD sense) and 5'-GTACGTGGAATAGAGCTTGG-3'; (GPI-PLD antisense) for GPI-PLD, and 5'-TGGTATCGTGGAAGGACTCATGAC-3' (actin sense) and 5'-ATGCCAGTGAGCTTCCCGTTCAGC-3' (actin antisense) for actin.

### Preparation of total lysates and caveolin-enriched membrane (CEM) fractions

Total brain lysates from the control and scrapie-infected mice (sampled at 60–160 dpi) were obtained by homogenization in lysis buffer [40 mM Tris (pH 8.0), 0.1% Nonidet P-40, 120 mM NaCl, 10 μg/ml leupeptin] in the presence of protease inhibitors (10 μg/ml leupeptin, 2 μg/ml aprotinin, and 1 mM phenylmethylsulfonyl fluoride). The CEM fractions were prepared as described previously [17], with some modifications. Briefly, the brains were homogenized with 100 strokes of a Dounce homogenizer in MES-buffered saline (MBS, 25 mM MES, pH 6.5, 150 mM NaCl). The homogenates were adjusted to 40% sucrose by the 1:1 addition of 80% sucrose prepared in MBS buffer and then placed in the bottom of a tube. Four milliliters of 30% and 4 ml of 5% sucrose in MBS buffer were consecutively layered on the top of the homogenates, and the gradients were centrifuged at 39,000 rpm for 18 h in a SW41 rotor (Beckman Instruments, Fullerton, CA). One milliliter fractions were collected from the top, yielding a total of 12 fractions from control and 12 from scrapie-infected brain.

### Western blot analysis

The total lysates and CEM fractions containing 50 μg of total proteins were subjected to 8–12% sodium dodecyl sulfate–polyacrylamide gel electrophoresis (SDS-PAGE). The separated proteins were transferred to nitrocellulose membranes (0.45 μm Pharmacia Biotech, Piscataway, NJ). After blocking the binding of nonspecific protein with 5% skim milk (BD, Franklin Lakes, NJ) in TBS buffer for 1 h, the membranes were incubated with an antibody for either goat anti-GPI-PLD (1:1,000), mouse anti-10E4 (1:5,000) or mouse anti-caveolin1 (1:500) at 4°C overnight. After washing three times in TBS buffer containing 0.05% Tween-20, the membranes were incubated for 1 h with horseradish peroxidase (HRP)-conjugated anti-rabbit IgG (Pierce, Rockford, IL), anti-goat IgG or anti-mouse IgG (Bio-Rad, Hercules, CA). The protein bands were detected by enhanced chemiluminescence (Pierce, Rockford, IL).

To detect PrP^Sc^, the brain homogenates (50 μg of protein) were treated with proteinase K (PK, 100 μg/ml) and then incubated for 30 min at 37°C prior to SDS-PAGE.

### Statistical analysis

Quantitative results were expressed as means ± standard deviations (SD), and the statistical significance between control and scrapie-infected mice was calculated using a Student's *t*-test.

## Results

### GPI-PLD mRNA and protein are down-regulated in the brains of scrapie-infected mice

**Fig 1:**
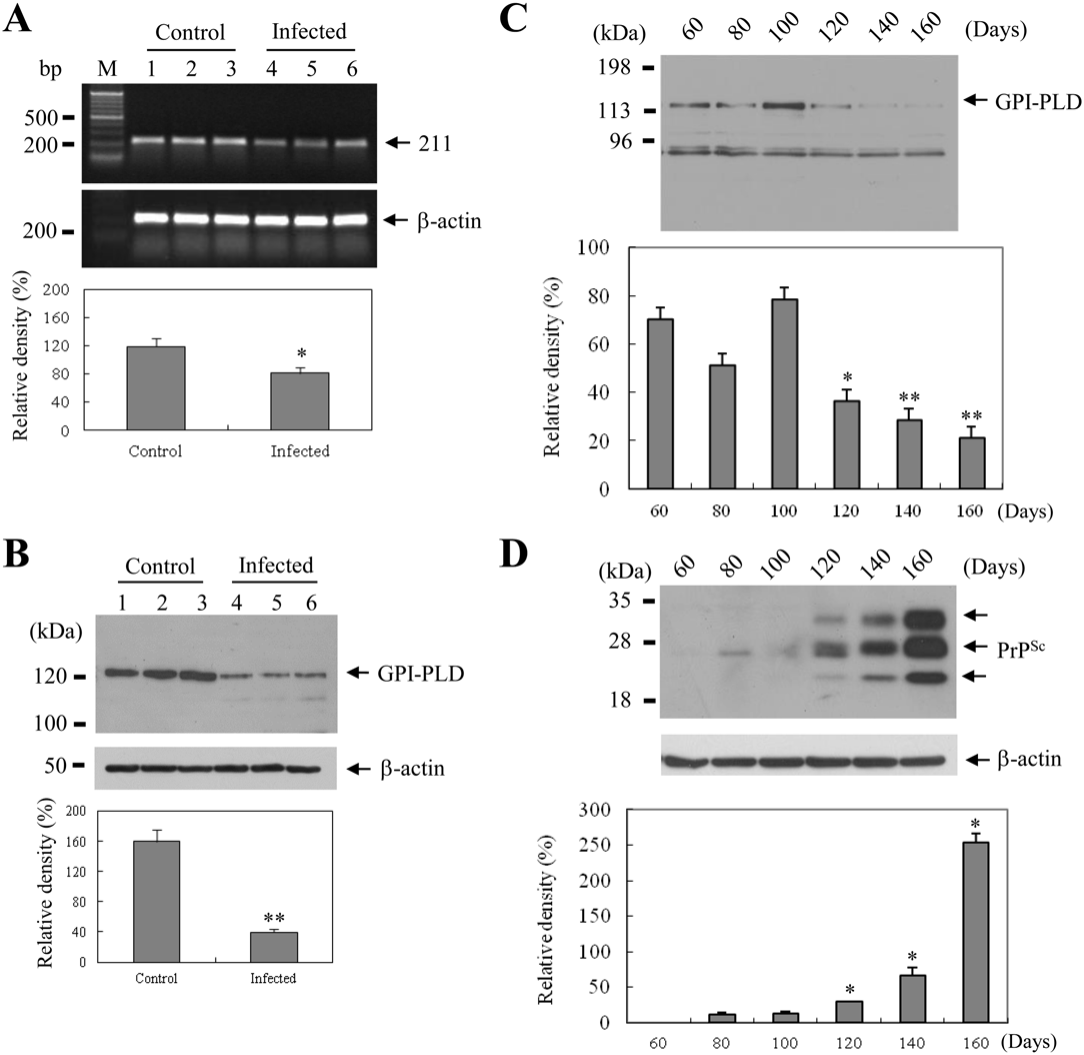
GPI-PLD is down-regulated in the scrapie-infected brains. (**A**) RT-PCR analysis of GPI-PLD expression levels using total RNA samples extracted from the whole brains of mice injected with either ME7 or normal brain homogenates at 160 dpi. The GPI-PLD mRNA levels were quantified using a densitometer analysis. Each value represents the mean ± SD of three samples. *Significance *P* < 0.05 compared with control levels. Actin was used as the control. (**B**) Western blot analysis of GPI-PLD protein levels in whole brain at 150 dpi from mice injected with either ME7 or normal brain homogenates. Total brain lysates of control and scrapie-infected mice were prepared as described in the *Materials and Methods* section and analyzed by western blotting with the GPI-PLD antibody. Each value represents the mean ± SD of three samples. **Significance *P* < 0.01 compared with control levels. Each experiment was repeated at least three times, and similar results were obtained in each experiment. β-actin was detected by anti-β-actin antibody and it served as the control. (**C-D**), GPI-PLD begins to be reduced at the time PrP^Sc^ is detected. Total lysates, which were prepared as described in the *Materials and Methods* section, were analyzed by Western blotting with the anti-GPI-PLD (C) and anti-PrP 10E4 antibodies; the latter experiment is done following PK treatment (D). Each experiment was repeated at least three times, and similar results were obtained in each experiment. β-actin was detected by anti-β-actin antibody and was used as the control. * Significance *p* < 0.05 compared to control 60 days after scrapie infection; ** Significance *p* < 0.01 compared to control 140 and 160 days after scrapie infection.

One hundred sixty days after the intracerebral injection of either ME7 or normal brain homogenate, we examined the GPI-PLD mRNA levels in control and scrapie-infected mouse brains using RT-PCR. As shown in Fig 1A, GPI-PLD mRNA was expressed strongly in control mice, but weakly in ME7 scrapie-infected mice. Because the GPI-PLD gene expression levels were decreased in the scrapie-infected brains at 160 dpi, the levels of GPI-PLD protein expression levels were examined in total brain lysates from control and scrapie-infected mice using Western blot analysis. The GPI-PLD protein was expressed strongly in the control brains, but weakly in the scrapie-infected brains (Fig 1B).

### The decrease in GPI-PLD expression levels is associated with PrP^Sc^ accumulation in scrapie-infected brains

The changes in GPI-PLD protein expression levels during disease progression of scrapie were examined in the total lysates of both control and scrapie-infected brains (sampled at 60~160 dpi). GPI-PLD expression levels showed a tendency to decrease as the disease progressed. In particular, Western blot analysis revealed that the GPI-PLD expression levels began to reduce at 120 dpi and decreased gradually during scrapie disease progression (Fig 1C). In contrast, the GPI-PLD expression levels were not changed as a function of time in control mice (data not shown). Next, we detected low amounts of abnormal prion protein (PrP^Sc^) at 120 dpi, and the level was increased gradually during scrapie disease progression (Fig 1D). These results suggest that the observed decrease in GPI-PLD protein levels may be closely related to PrP^Sc^ formation.

### Expression of GPI-PLD protein is decreased in the CEM fractions of scrapie-infected brain

**Fig 2:**
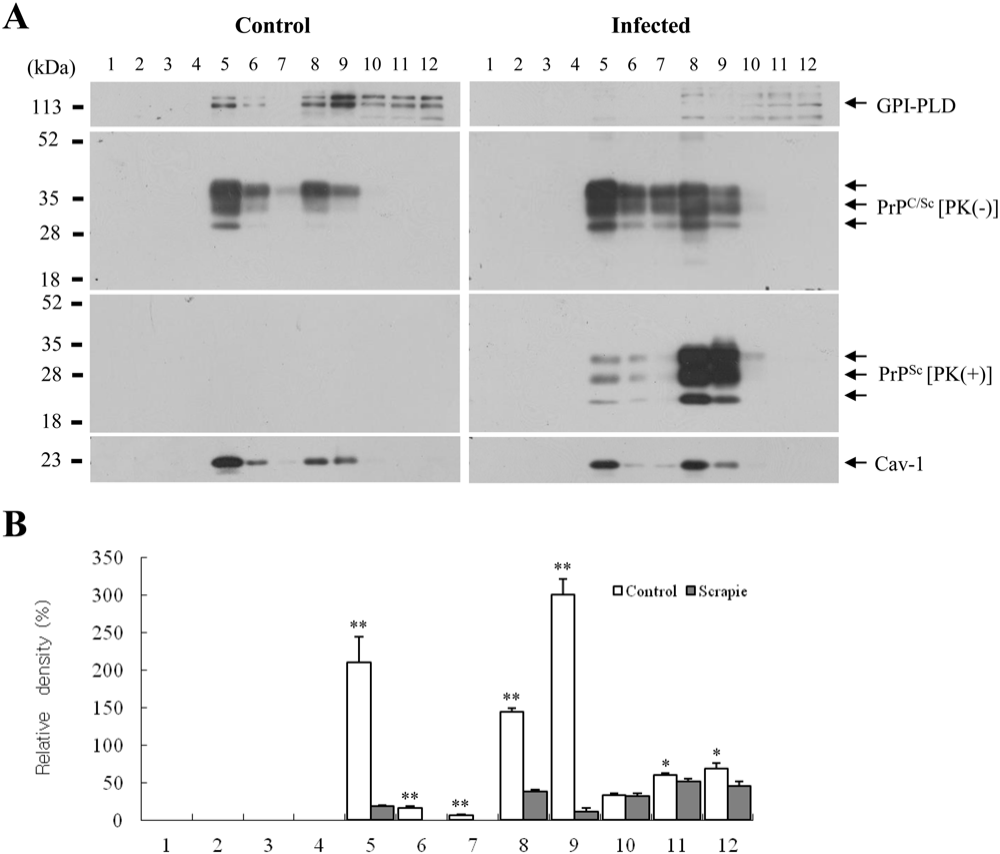
A decrease in GPI-PLD expression was observed in the caveolin-enriched membrane (CEM) fraction. Gradient fractions from control and from scrapie-infected brain lysates were assayed for GPI-PLD and PrP^Sc^ as described in the *Materials and Methods* section. Equal sucrose gradient fractions (1 ml each following centrifugation) were analyzed by Western blotting with the anti-GPI-PLD and anti-PrP 10E4 antibodies. Each experiment was repeated at least three times, and similar results were obtained in each experiment. An anti-caveolin-1 antibody was used as the positive control for the CEM fractions. Note lanes 5, 8 and 9, in which PrP^Sc^ and caveolin-1 are abundant. * Significance *p* < 0.05 compared with control levels; ** Significance *P* < 0.01 compared with control levels.

Next, we examined the expression level of GPI-PLD in the CEM fractions that were extracted from control and scrapie-infected brains. As shown in Fig 2A, high levels of GPI-PLD were found in control mice, yielding intense staining in lanes 5, 8 and 9 in CEM preparations. In contrast, there was virtually no staining in any fractions prepared from scrapie brains. The relative density of GPI-PLD is shown in Fig 2B. The gradient fractions in control mice that contain large amounts of GPI-PLD were shown to have high levels of PrP^Sc^ in gradient fractions from scrapie mice (Fig 2A). These results suggest that the decreased expression of GPI-PLD in scrapie-infected brains is correlated with PrP^Sc^ formation during scrapie disease progression.

### The level of GPI-PLD expression is decreased in both the brains and CSF of CJD patients

**Fig 3:**
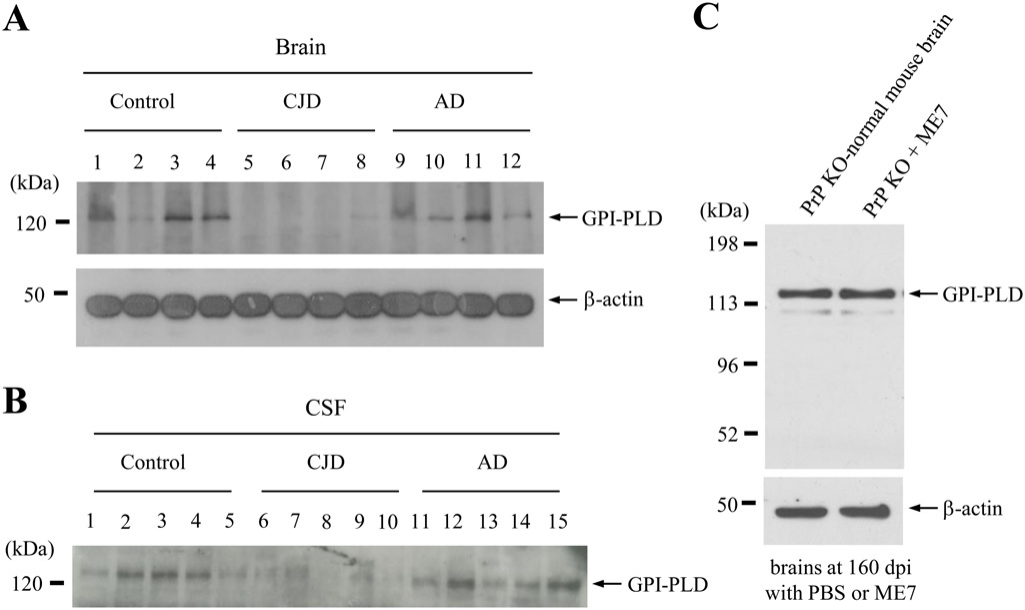
A decrease in GPI-PLD levels was observed in the brains and CSF specimens of CJD patients. Compared to controls, the level of GPI-PLD expression was decreased in brains (A) and CSF specimens (B) from CJD patients. In contrast, a change in GPI-PLD expression levels was not observed in the brains of either AD or normal/scrapie strain (ME7)-injected PrP knock out (PrP KO) (C) mice. All brain tissues were prepared as described in the *Materials and Methods* section and analyzed by Western blotting using the anti-GPI-PLD and anti-PrP 10E4 antibodies. All brains and CSF specimens shown in Table 1 were analyzed by Western blotting. β-actin detected by an anti-β-actin antibody was used as the control.

Next, we examined the expression levels of GPI-PLD in the brains (Fig 3A) and CSF (Fig 3B) specimens from both AD and CJD patients. Interestingly, the down-regulation of GPI-PLD protein expression was observed in both the brains and CSF obtained from CJD patients. Compared with normal controls, no difference in GPI-PLD expression levels was observed in either the brains or CSF specimens from AD patients (Fig 3A and 3B). Finally, 160 days after inoculation with either the ME7 scrapie strain or control inoculum, we determined the level of GPI-PLD expression in the brains of PrP knockout mice, which lack the Prnp gene. There was no difference in the pattern of GPI-PLD expression in PrP^C^-negative (knock-out) compared to PrP^C^-positive controls (Fig 3C).

## Discussion

The major findings of the present study were that the GPI-PLD protein was dramatically downregulated in the brains of both a prion animal model and in human CJD patients, and that the GPI-PLD protein levels were especially decreased in the caveolin-like domains in which PrP^Sc^ is primarily found during prion propagation.

The conformational change of PrP^C^ is the most critical factor in the pathogenesis of prion diseases: the mechanisms involved in prion protein conversion *in vitro* have been extensively investigated. Nucleic acids, an acidic pH and transition metals may influence the cell-free conversion of prion, in which the purified PrP^C^ and the denatured PrP^Sc^ are mixed *in vitro* [18, 19]. However, few *in vivo* studies of the mechanisms by which PrP^C^ is converted to PrP^Sc^ have been conducted. In general, PrP^C^ is primarily localized in the CLDs of neuronal cells where the conformational change from PrP^C^ to PrP^Sc^ occurs [5]. Consistent with the above report, in the present study PrP^Sc^ was predominantly observed in the caveolae and CLDs. For this reason, we speculated that the pathogenic mechanisms of prion disease may be influenced by the environment of the caveolin-enriched membrane. It has been well documented that the cellular signaling of GPI-anchored proteins, including prion protein, in the caveolin-enriched membrane compartment is regulated by PI-PLC. PI-PLC may play an important role in modulating the cell surface expression and function of GPI-anchored proteins [20]. Recent *in vitro* studies have demonstrated that PI-PLC treatment can inhibit proteinase k-resistant PrP (PrP^res^) formation in scrapie-infected neuronal cell lines (ScN2a cells) [21, 22]. In contrast, the role of GPI-PLD, one of the secreted mammalian enzymes that specifically cleave GPI-anchored proteins, has not been reported in prion disease.

In a previous report, PrP^C^ was associated with caveolin, a structural protein component of the caveolae [23]. Because the function of GPI-PLD remains unclear, the role of decreased GPI-PLD expression levels must be elucidated in the scrapie-infected brain. One possibility is that a decrease in GPI-PLD, which cleaves the phosphatidylinositol in GPI-anchored proteins, may induce a reduction in the release of the cellular prion protein, and, in turn, deposit the converted PrP^Sc^ into the plasma membrane, a major pathologic hallmark of prion diseases, including scrapie. Increasing evidence suggests that GPI-PLD induces the release of GPI-anchored proteins in HeLa and bone marrow stromal cells [24, 25]. Scallon *et al* [11] and Bernasconi *et al* [26] reported that anchored proteins were released by the co-transfection of GPI-PLD and GPI-anchored proteins in COS-1 cells. Based on this evidence, we propose that the conformational change from PrP^C^ into PrP^Sc^ occurs in the CEM and that this event may be the result of decreased levels of GPI-PLD. It is currently unclear whether the down-regulation of GPI-PLD is involved in PrP^Sc^ formation or in oxidative stress. Further experiments must be performed to clarify the specific role of GPI-PLD in the brains of prion diseases.

The exact mechanisms involved in the down-regulation of GPI-PLD are unknown. A recent study reported that the decrease in GPI-PLD was regulated by lipopolysaccharides and oxidative stress in the murine monocyte-macrophage cell line and that the down-regulation of this enzyme can play an important role in the control of proinflammatory responses [27]. In this context, GPI-PLD may be involved in modulating the inflammatory process during scrapie disease. However, there is little evidence of inflammatory cell migration in scrapie disease. Previously, our laboratory has provided evidence that oxidative stress can be detected in the brains of mice infected with the 87V scrapie strain [28], in hamster brains infected with the 263K scrapie strain, mitochondrial dysfunction was induced via an increase in lipid peroxidation, which is believed to be one of the mechanisms of cellular damage [29]. In a recent study, we reported that phosphatidic acid (PA) contents were significantly increased in scrapie-infected brains [30]. In addition, plasma GPI-PLD has been reported to be inhibited by PA and lysophosphatidic acid (LPA) [31, 32]. PA generated by the action of Phospholipase D has been known to act as an intracellular second messenger[33] and can be converted to other messenger molecules such as 1, 2-diacylglycerol and lysophosphatidic acid (LPA) [34]. The down-regulation of GPI-PLD in scrapie-infected brains may be regulated by the generation of phospholipid products, PA and LPA, which, in turn, may play a role in the development of neurodegeneration in prion disease. Thus, we hypothesize that the GPI-PLD down-regulation by oxidative stress and that PA may contribute to a co-factor that is allegedly involved in the conversion of PrP^C^ into PrP^Sc^.

In conclusion, the findings from the present study suggest that the down-regulation of GPI-PLD inhibits the release of the GPI-anchored prion protein (PrP^C^) from the CEM, resulting in more interaction with the injected scrapie prion protein (PrP^Sc^) as the disease progresses. Thus this observation opens the possibility that the GPI-PLD down-regulation in the scrapie-infected brains can influence the post-translational conversion of PrP^C^ into the scrapie isoform (PrP^Sc^) and accelerate PrP^Sc^ formation in the end stage of prion disease. To clarify the specific mechanisms of prion conversion in prion disease, experiments using *in vitro* and transgenic animal models are being conducted.
